# Functional end-arterial circulation of the choroid assessed by using fat embolism and electric circuit simulation

**DOI:** 10.1038/s41598-017-02695-z

**Published:** 2017-05-30

**Authors:** Ji Eun Lee, Ki Su Ahn, Keun Heung Park, Kang Yeun Pak, Hak Jin Kim, Ik Soo Byon, Sung Who Park

**Affiliations:** 1 Department of Ophthalmology, College of Medicine, Pusan National University, Yangsan, Korea; 2 Biomedical Research Institute, Pusan National University Hospital, Busan, Korea; 3 Jung Eye Clinic, Geoje, Korea; 4 Department of Radiology, College of Medicine, Pusan National University, Yangsan, Korea; 5 Research Institute for Convergence of Biomedical Science and Technology, Pusan National University Yangsan Hospital, Yangsan, Korea

## Abstract

The discrepancy in the choroidal circulation between anatomy and function has remained unsolved for several decades. Postmortem cast studies revealed extensive anastomotic channels, but angiographic studies indicated end-arterial circulation. We carried out experimental fat embolism in cats and electric circuit simulation. Perfusion defects were observed in two categories. In the scatter perfusion defects suggesting an embolism at the terminal arterioles, fluorescein dye filled the non-perfused lobule slowly from the adjacent perfused lobule. In the segmental perfusion defects suggesting occlusion of the posterior ciliary arteries, the hypofluorescent segment became perfused by spontaneous resolution of the embolism without subsequent smaller infarction. The angiographic findings could be simulated with an electric circuit. Although electric currents flowed to the disconnected lobule, the level was very low compared with that of the connected ones. The choroid appeared to be composed of multiple sectors with no anastomosis to other sectors, but to have its own anastomotic arterioles in each sector. Blood flows through the continuous choriocapillaris bed in an end-arterial nature functionally to follow a pressure gradient due to the drainage through the collector venule.

## Introduction

The choroid is a very special tissue, as it has the most abundant blood flow per weight in the human body and a highly complicated three-dimensional vascular structure. Choroidal circulation has been suggested to be predisposed to many diseases, including age-related macular degeneration^1, 2^, polypoidal choroidal vasculopathy^3^, central serous chorioretinopathy^4^, and idiopathic choroidal neovascularization^5^. However, many aspects of choroidal circulation are still unknown.

A majority of our knowledge about choroidal circulation has been obtained from classical studies using autopsy and angiography. Postmortem corrosion cast studies revealed that choroidal circulation has anastomotic channels at various levels of capillaries, arterioles, and venules^6, 7^. These characteristics are shared with various mammals including monkeys^6^, horses^8^, and cats^9^. In contrast, extensive angiographic studies conducted *in vivo* by Hayreh^10–12^ indicated that the choroid has an end-arterial system. Recent advent of choroidal imaging using new technologies, such as Doppler optical coherence tomography and continuous laser-targeted angiography, affirmed the previous observations, leaving the disparity unresolved^13–15^. Although an autonomic nerve supply of the choroid *in vivo* was suggested as explanation, the discrepancy between the anatomy and the function has remained unsolved for several decades^10, 12^.

Flower and colleagues^16, 17^ suggested that the blood follows a pressure gradient *in vivo* based on the observations that choriocapillaris blood flow was inhomogeneous and variable upon changes in blood pressure and intraocular pressure. Although the vascular channels are interconnected in the choroid, the blood will follow pressure gradient and will be observed as an end-arterial system in angiography. A “functional end-arterial system” represents the above theory^15^. However, the theory has not been proven, in part because generation of a crossing flow upon reversal of the pressure gradient has not been observed. Moreover, the sectorial non-perfusion observed in the occlusion of the posterior ciliary artery (PCA) cannot be explained by their theory, as the pressure gradient in the choroidal arterioles will be reversed when the PCA is occluded, and the non-perfusion would be resolved rapidly by perfusion via the anastomosing arterioles from the adjacent sectors.

Triolein emulsion was used for an experimental fat embolism model^18, 19^. Infusion of triolein emulsion into the carotid artery causes microembolism in the intraocular circulation, and has several advantages in the investigation of the microcirculation of the eye. A triolein emulsion can have various particle sizes, which result in different findings, as vessels of various sizes were occluded. This allowed the observation of both sectorial and lobular perfusion defects. Another important feature is that triolein is liquid, and thus, embolization can be readily resolved. Angiographic findings can be recorded at the moment when embolism is resolved^19^. The feline eye has a special layer, the tapetum lucidum cellulosum, between the choriocapillaris and the larger choroidal vessels^20^. This layer reflects incident light back to the photoreceptor and increases light perception in darkness. In addition, the dense pigmentation of the layer blocks fluorescence from the large choroidal vessels, which were not demonstrated even in indocyanine green angiography in our preliminary study. Thus, we decided to use fluorescein angiography (FA) to obtain higher-contrast images. The use of FA in feline eyes has the advantage of assessing the choriocapillaris circulation without interfering with background fluorescence.

Meanwhile, blood circulation may be demonstrated schematically using an electric circuit, with the electric current corresponding to flow, voltage to pressure, and electric resistance to vascular resistance^21^. This model would be helpful in simplifying a complicated vascular network and provide explanation for the circulation changes observed in experiments as well as in pathologic conditions.

We investigated the choriocapillary circulation using FA in an experimental fat embolism model, and the angiographic findings were interpreted by using an electric circuit model simulating the choriocapillary bed.

## Results

### Choroidal circulation in the fat embolism model

Various degrees of perfusion defects were demonstrated in the retina and choroid. The difference in degrees was due to the various sizes of triolein emulsion droplets^18^. Stop-and-go movements of intravascular lipid drops were observed in the retinal vessels, and the perfusion defects were resolved in most areas in the late phase.

**Figure 1:**
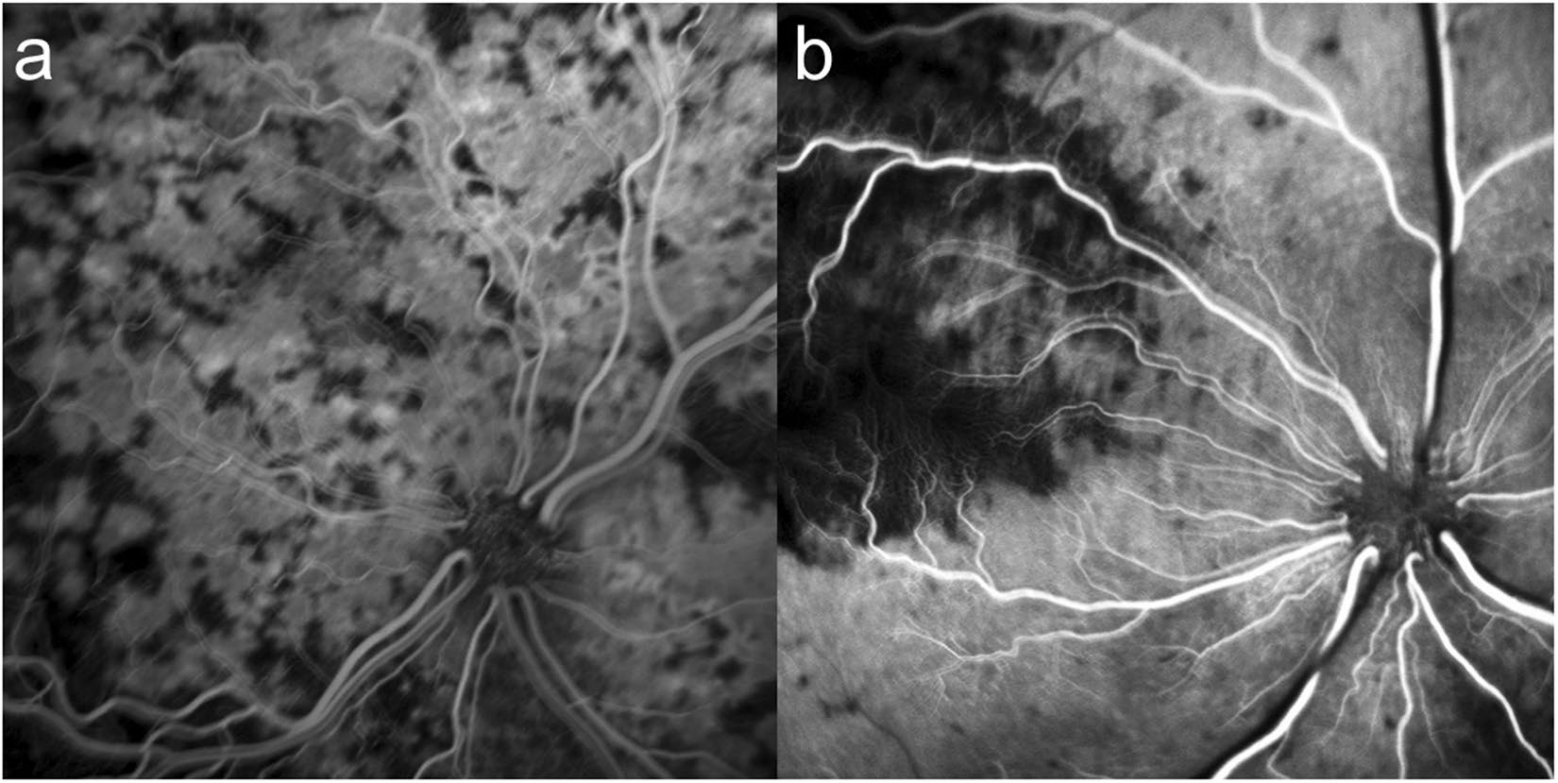
Two types of perfusion defects in the choroid were observed in the fat embolism model by using triolein emulsion. (**a**) Multiple patch defects indicate that arterioles feeding the choriocapillary lobule are occluded. (**b**) Segmental hypofluorescence indicates that the short posterior ciliary artery supplying the segment is embolized.

The perfusion defects of the choroid were observed in two categories (Fig. 1): (1) perfusion defects scattered among small choroidal patch fillings in 8 cats and (2) segmental perfusion and defects assumed to correspond to territories supplied by the PCAs in 4 cats.

**Figure 2:**
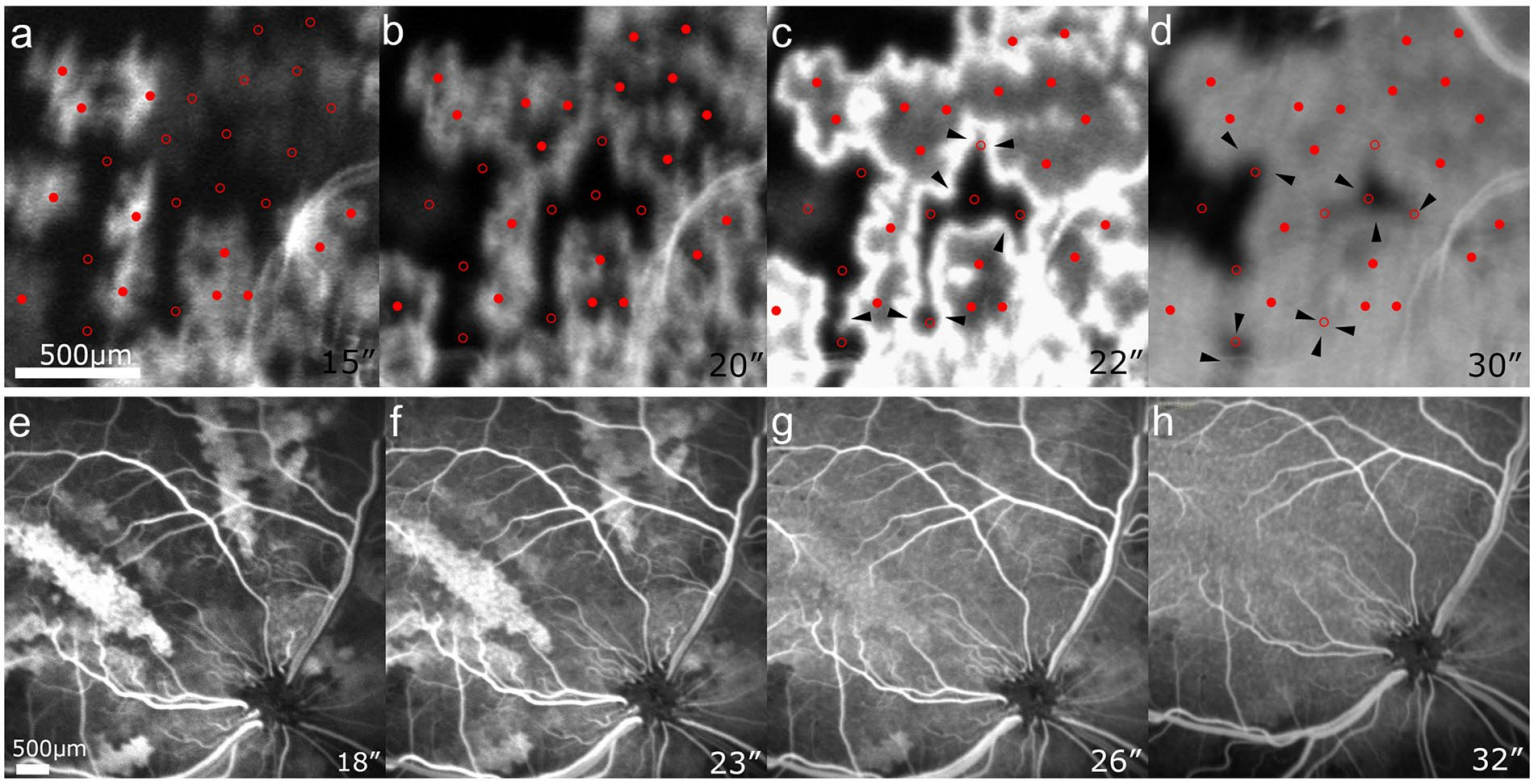
Fluorescein angiography obtained in cat eyes after triolein fat embolism. (**a**–**d**) Multiple hyperfluorescent and hypofluorescent patches are observed, representing perfused and embolized choriocapillaris lobules. Red dots and circles represent the locations of the perfused and embolized terminal arterioles, respectively. The expansion of the hyperfluorescent area clearly demonstrates the blood flow beyond the boundary of each lobule (arrowheads). (**e**–**h**) Sectorial hyperfluorescence and hypofluorescence represent the occlusion of the short posterior ciliary arteries. After approximately 10 seconds, perfusion defects were resolved without any subsequent defects.

In the former, the small choroidal patches were filled with fluorescein dye at multiple locations in a centrifugal pattern from the center of each location, representing lobular perfusion of the choriocapillaris. All the centers of the lobules could not be marked in areas having broad and prolonged non-perfusion. The distance between two adjacent feeding arterioles, measured based on the points where hyperfluorescence began to show in each lobule, was approximately 200 to 400 μm. The scattered perfusion defects suggested a fat embolism at the terminal arterioles supplying the lobules. Some defects were resolved as the dye began to fill the choriocapillaris centrifugally from the lobular center, conversely some of the perfused lobules became non-perfused due to delayed embolism (Fig. 2a–d, Supplemental Video). As reported previously, various findings were noted related to various particle size of triolein emulsion. Irregular initial filling of the lobule was observed, probably because of triolein emulsion in the choriocapillaris. Filling of fluorescein was slow and sometimes creeping. Reperfusion of several lobules in a group was also noted.

Notably, dye filling from the perfused lobule to the adjacent non-perfused lobule was clearly observed, indicating that the choriocapillaris is not an end-arterial system anatomically. However, the filling rate was so slow that a non-perfused lobule surrounded by perfused lobules was barely filled after approximately 10 seconds.

In the latter, the perfused and non-perfused segments were demonstrated as discrete hyperfluorescent and hypofluorescent areas, respectively (Fig. 2e–h). The boundaries remained almost unchanged, with minimal spread of fluorescence for 12 seconds in average (range; 9–15 s). Then, the hypofluorescent segment was perfused, indicating spontaneous resolution of the triolein embolism, and the segments became indiscernible (Fig. 2h). Subsequent non-perfusion of the smaller segments or flow of the released triolein was not observed.

### Interpretation using electric circuit simulation

**Figure 3:**
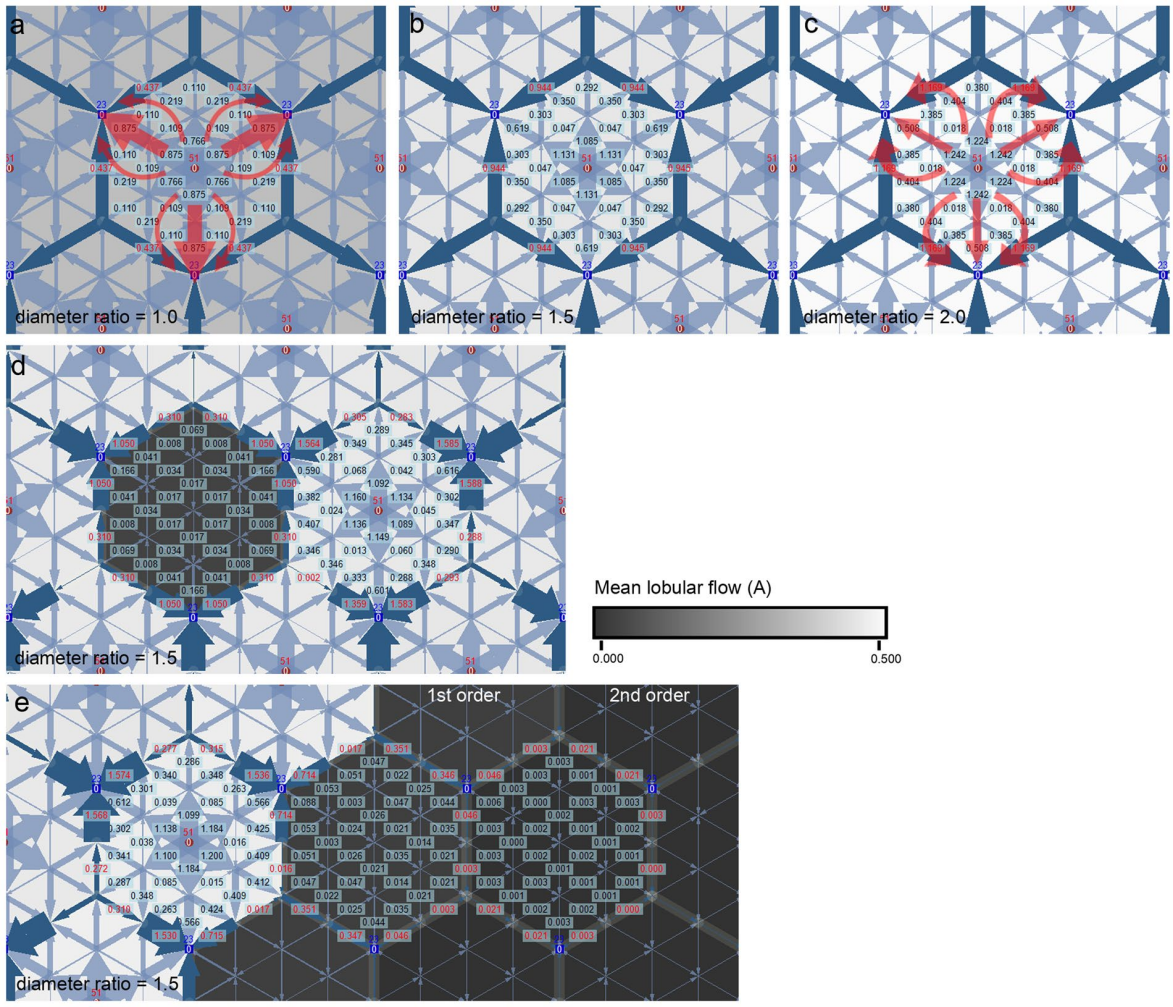
Electric circuit simulation of the choroid. (**a**–**c**) The normal choroid shows that electric currents follow the voltage gradient and do not cross the boundary of the hexagon. As the resistance of the boundary becomes lower, the blood flows become more evenly distributed in the hexagon. (**d**) When one anode is disconnected from the hexagon, the voltage gradient is reversed. Electric currents flow from the adjacent hexagons and cross the boundary to the disconnected hexagon, but at very low level. (**e**) Reduction of electric current is more severe in sectorial disconnection. Diameter ratio indicates ratio of the collector venule at the lobular boundary and the choriocapillaris. Electric resistance is inversely proportional to the fourth power of the radius (r^4^) by Poiseuille’s equation.

In the normal circuit model, the direction of the electric currents was centrifugal from the anodes toward the periphery of the hexagon and centripetal around the cathodes to follow the voltage gradient (Fig. 3a–c). The electric current from adjacent hexagons converged in the boundaries and flowed along the boundaries to the cathodes. No current crossing the boundaries of the hexagon was observed. When the resistance of the hexagon boundary was set at 16 Ω, representing the collector venule with the same diameter as the capillary, the direct currents were dominant from the anode to the cathodes in the hexagon. As the resistance of the hexagon boundary decreased from 16 to 1 Ω, the average current of a hexagon increased, and electric currents were distributed more evenly in the hexagon.

**Figure 4:**
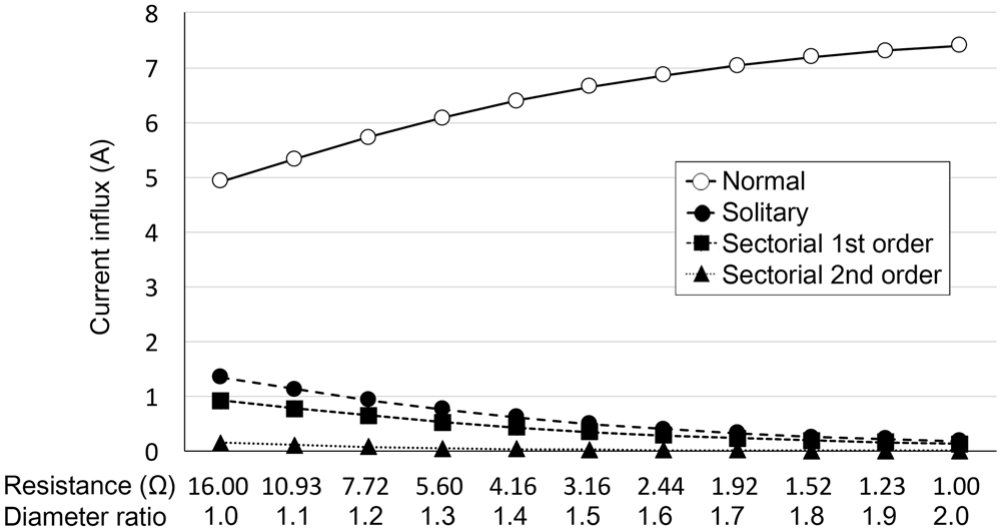
Changes in electric current influx into a hexagon due to change in boundary resistance. When the resistance of the boundary is lower, more currents flow normally, but to a lesser degree in the disconnected hexagons.

When a single anode was removed in the single feeding arteriole embolism model, electric currents were rearranged. As the voltage gradient from the hexagon center was reversed, electric currents flowed to the disconnected hexagon from the adjacent hexagon, crossing the boundary (Fig. 3d). However, the influx to the disconnected hexagon decreased dramatically to 27.3–2.5% of the anode-connected hexagon depending on the resistance of the boundary. Circuits with lower boundary resistance had lower currents to the disconnected hexagon when disconnected (Fig. 4).

In the model of ciliary artery occlusion, the electric currents in the disconnected hexagons were even lower than in the solitary disconnection condition (Fig. 3e). The electric influx in the first-order hexagons in the disconnected sector reduced to 18.6–1.8% of the connected hexagon, whereas that of the second-order hexagons had electric influx reduced to 2.8–0.04% (Fig. 4).

## Discussion

Our study revealed that the choriocapillary lobule was perfused *via* capillary anastomosis from the adjacent lobule *in vivo*, which has never been observed in previous studies. The method different from that used in previous studies enabled to find the novel results in the present study.

Many angiography studies in animals demonstrated that the choroid has an end-arterial system, and the use of FA in rhesus monkey showed a mosaic pattern of choriocapillary perfusion^22^. A study using continuous laser-targeted angiography also demonstrated that the fluorescence did not spread outside of the choroidal lobule^15^. The above experiments were performed in eyes with normal circulation. Our electric circuit model simulating normal circulation also revealed that electric currents flow as a unit of hexagon with no currents crossing the boundary, as the voltage gradient was formed in each lobule from the central anode to the boundary and the cathodes. The currents were higher near the cathode and anode, and showed inhomogeneity consistent to findings in previous theoretical models^23, 24^. If this model is applied to the choriocapillaris in the interpretation of *in vivo* observations, blood flows from the feeding arteriole to the collector venule and then to the draining venules in each lobule and will not flow to the adjacent lobules.

Although angiographic findings of triolein embolism in cat eyes were diverse due to various lipid size of triolein emulsion and complexity of the choroidal vasculature, we identified the following noteworthy observations. When triolein emulsion embolized a feeding arteriole to a lobule, the flow of fluorescent dye was observed from the adjacent lobules to the embolized lobule. The hyperfluorescent lines seen in Fig. 2b and c were different from the mosaic pattern observed in the experiment using bolus injection of fluorescein dye^22^, and did not indicate the borders of the choroidal lobules. In our experiment, fluorescein dye was not injected as a bolus, and the hyperfluorescent lines suggest stasis of the flow due to triolein emulsion. Between the perfused lobules, the bright lines were not demonstrated, as fluorescein dye was drained through the draining venule. However, once fluorescein dye flowed into the non-perfused lobule, it would not be easily washed out. Although the choriocapillaris has fenestration and triolein may increase leakage by opening the blood-retinal barrier, the spread of the fluorescein dye to the center of the embolized lobule appeared to be much faster than the diffusion. In addition, diffusion follows the concentration gradient, and spread of fluorescein by diffusion should have the frontier of less fluorescence then the source. Accordingly, the pattern of spreading fluorescence in Fig. 2b–d indicates that filling of the choriocapillaris perfused the embolized lobule.

This could also be simulated using the electric circuit model by removing an anode connection from a single hexagon. As the voltage gradient from the lobular center was reversed, electric currents were formed from the adjacent hexagons. However, the currents decreased to a very low level compared with an anode-connected hexagon. Especially around the anode, the electric current dropped to about 1% level of the perfused (Fig. 3d). When changing the boundary resistance, circuits with lower resistance had higher currents in the connected hexagon and lower currents in the disconnected hexagon. Applying the simulation results to the choriocapillaris circulation indicates that a more efficient drainage *via* the choroidal venule results in more reduction of blood flow to the lobule when embolized. The profound reduction in blood flow seems sufficient in causing focal ischemic changes in the choroid, such as Elschinig’s spot and acute posterior multifocal placoid pigment epitheliopathy, despite a potentially redundant flow.

Both angiographic studies in triolein embolism and electric circuit simulation demonstrated results corresponding to the occlusion of a PCV or its branches. However, a discrepancy remained because anastomosis among the choroidal arterioles was depicted in postmortem studies^25, 26^. Autonomic regulation *in vivo* ^12^ or development of quick collaterals^11^ has been suggested as an explanation for the discrepancy. The most interesting finding of the present study was the spontaneous resolution of the short PCA occlusion, as subsequent embolization of smaller vessels was not observed. These results cannot be explained by autonomic nerve regulation or collateral formation. If a sector of the choroid has no established anastomosis among the arterioles, a drop of triolein released from a large artery will flow down to smaller arterioles or capillaries, which should be embolized and show up as a perfusion defect in angiography. Even if a drop is broken into small droplets which flow without embolism, they should be observed as a flow of fragmented fluorescence^18^. However, such a flow of triolein was not observed either.

**Figure 5:**
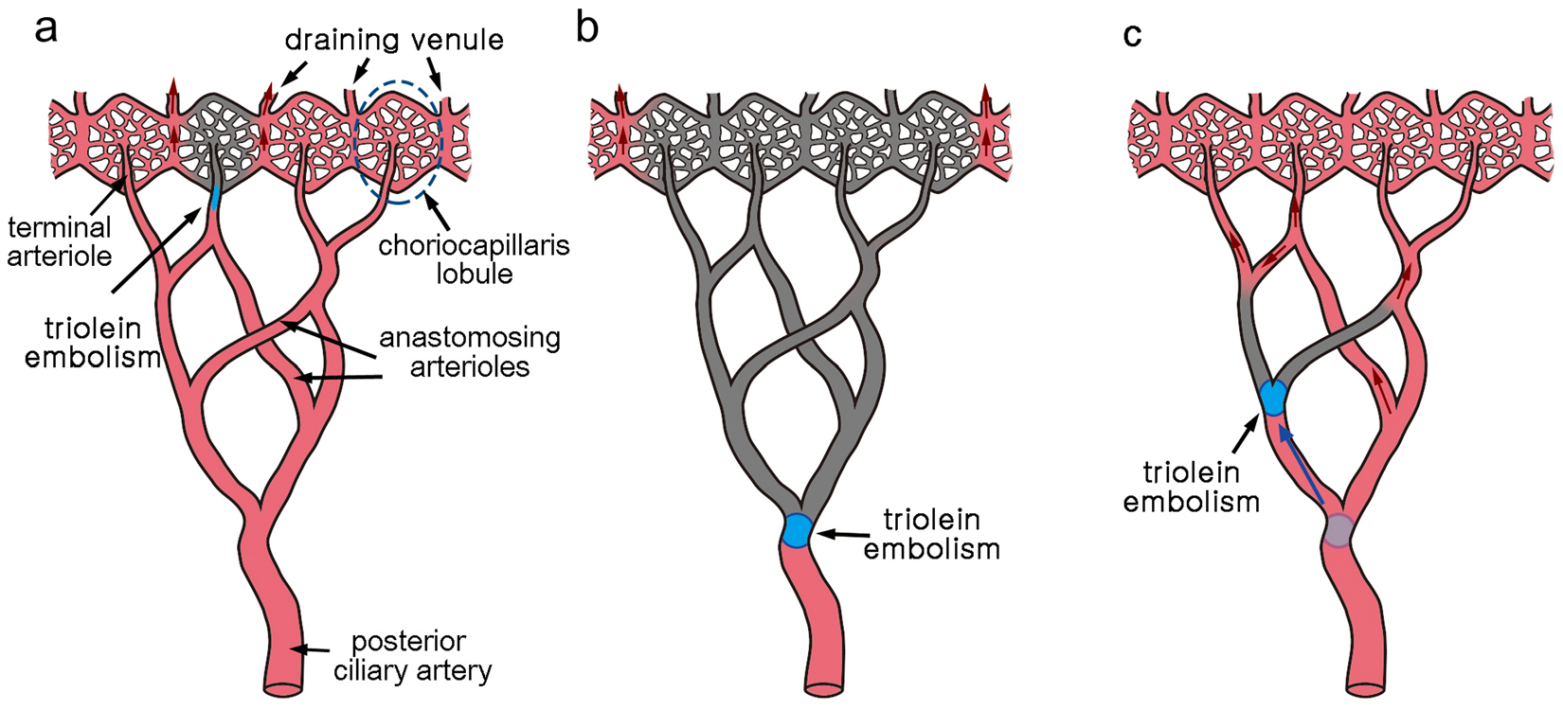
A functional end-arterial model of the choroidal circulation. Red and gray vascular channels represent perfused and non-perfused status, respectively. (**a**) When a terminal arteriole is obstructed, the choriocapillaris lobule becomes ischemic because the blood is drained through the venous channel in the periphery of the lobule. (**b**) A sector becomes ischemic when the posterior ciliary artery is occluded, as there is no arteriolar anastomosis among the other sectors. (**c**) When triolein embolus flows down to a small arteriole, perfusion of the choriocapillaris is restored owing to the extensive arteriolar anastomosis within the sector.

Based on the above observations, we suggest a new model for the choroidal vascular pattern (Fig. 5). The choroid is composed of multiple sectors with no anastomosis to other sectors, but each sector has its own anastomotic arterioles. Itotagawa *et al*. reported an example in the second figure of the literature^6^. The postmortem cast demonstrated that two anastomosing vessels had the common trunk. The choriocapillaris has a continuous vascular bed, but the blood flows in an end-arterial nature because of drainage to the collector venule at the periphery of each lobule. In this vascular pattern, occlusion of a terminal arteriole and a PCA results in a lobular (Fig. 5a) and sectorial infarction (Fig. 5b), respectively. By contrast, when a small choroidal arteriole is occluded by triolein emboli released from the distributing PCA, choroidal perfusion will be restored *via* anastomosis (Fig. 5c). For an example, experimental occlusion of medial PCA or lateral PCA resulted in a perfusion defect of the area supplied by the PCA^27^. These findings can be explained as the segments supplied by the PCA do not have anastomosis with each other, but may have anastomosis within each segment. Our model proposes that the choroidal circulation is functionally end-arterial not only in physiologic conditions but also in pathologic conditions, and may explain the results from the present and previous studies^15, 25–27^ without disparity.

One of the limitations of our study was that the large choroidal vasculature was not visualized directly using angiography or postmortem cast study. Occlusion of the large vessels may have affected the perfusion of the choriocapillaris. In addition, the arteriolar structures in the suggested model were based on the interpretations of the studies for the choriocapillaris. Our model should be verified in future studies, as understanding the choroidal circulation is essential in the investigation of its role in both physiologic and pathologic conditions.

In summary, our results suggest that the findings of the postmortem study directly correspond with the angiographic findings and that the discrepancy was a misunderstanding. The choroid is composed of multiple sectors, each having anastomotic arterioles within the sector. Blood flows through the continuous choriocapillaris bed in an end-arterial nature functionally to follow a pressure gradient, as efficient drainage through the collecter venules prevents flow from crossing the boundary among the choroidal lobules. These structures of the choroid substantiate its vulnerability to ischemic damages in spite of existing anastomosis.

## Methods

### Fat embolism model

FA was acquired in the previous experiment of fat embolism using a triolein emulsion to assess the blood-ocular barrier breakdown^19^. The study design was approved by Institutional Animal Care and Use Committee (IACUC) at Pusan National University Hospital (2012–038), and the procedures were performed in adherence to the Institutional Guidelines.

In brief, 12 adult cats weighing 2.5–3.7 kg were anesthetized with intramuscularly administered ketamine HCl (2.5 mg/kg; Korea United Pharm, Seoul, South Korea) and xylazine (0.125 mg/kg; Bayer Korea, Seoul, South Korea) and ventilated with room air. Their body temperature was monitored with a rectal probe (MGA-III219; Shibaura Electronics, Tokyo, Japan) and maintained at 35–36 °C. The right femoral artery was isolated under anesthesia. A 3.0-F microcatheter (MicroFerret-18 infusion catheter; William Cook Europe, Bjaeverskov, Denmark) was passed through an 18-gauge catheter inserted into the femoral artery. The tip of the microcatheter was positioned in the internal carotid artery under digital subtraction angiography. The triolein emulsion was prepared by mixing neutral triglyceride triolein (1,2,3-tri[*cis*-9-octadecenoyl] glycerol; Sigma, St. Louis, MO, USA) and normal saline with vigorous to-and-fro movements. The emulsified triolein (2.2%, 10 mL) was infused into the internal carotid artery at a rate of 4 mL/min. Fluorescein dye (0.3 mg/kg) was injected through the femoral vein immediately after the infusion of the triolein emulsion. Video FA was recorded in the ipsilateral eye at variable frame rate up to 16 Hz for approximately 30 seconds after the injection of fluorescein using a scanning laser ophthalmoscope (HRA2; Heidelberg Engineering, Heidelberg, Germany). FA photographs were obtained in both eyes at a rate of one frame per 2–3 seconds for 1 minute and then every 5 minutes for up to 30 minutes.

The locations of the terminal arterioles were marked based on the perfusion pattern observed in video FA. A terminal arteriole was identified to be located at where centrifugal filling and wash-out of fluorescein dye began. In some of non-perfused lobules, the center of the lobule was presumed based on the filling pattern of the surrounding lobules. Each location was overlapped in FA images, and was marked as a red dot in the perfused lobule and as a red circle in the non-perfused.

### Electric circuit model

The choriocapillaris was simulated as a schematic electric circuit using the custom software to interpret the angiographic findings. Blood flow was modeled as electric current (I), intravascular pressure as voltage (V), and vascular resistance as electric resistance(R)^21^.

The custom software was developed with Microsoft Visual Studio 2012 and C# language with. NET Framework (Microsoft, Redmond, WA, USA). To analyze the complicated electric circuit, we used Kirchhoff’s current law, which is the basis of most modern circuit analyzer software. Kirchhoff’s current law states that the total sum of in- and out-current is always zero at every point where there is a confluence of electric currents.

**Figure 6:**
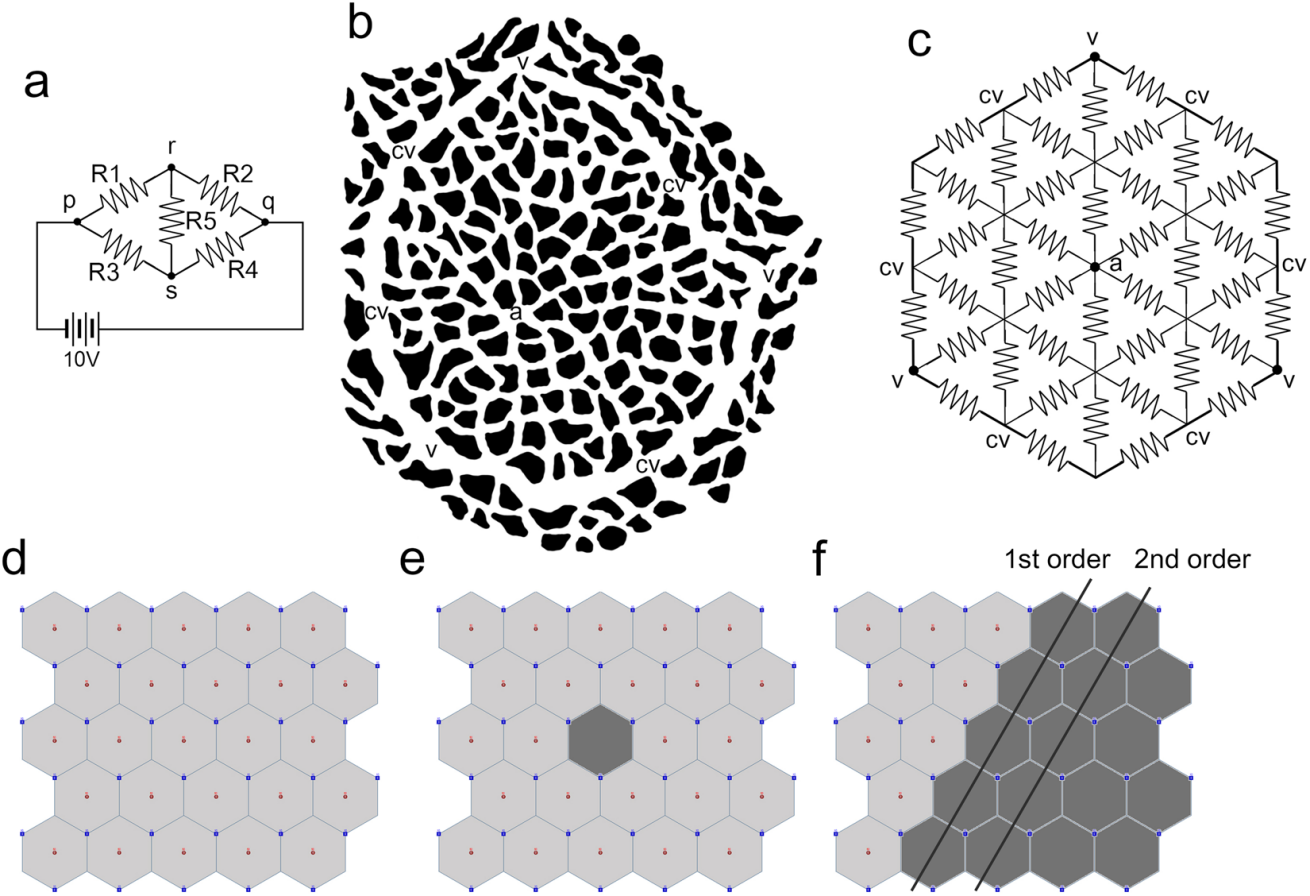
Schematic drawings of the choroidal lobule and its electric circuit model. (**a**) A simple example to explain Kirchhoff’s law. (**b**) The choriocapillaris of the posterior pole is arranged in a lobular pattern surrounded by the collector venules (cv). The feeding arteriole (*) joins the choriocapillaris at the center of the lobule. The draining venules (dv) are connected to the periphery of the lobules. (**c**) The electric circuit model of the choroidal lobule is arranged as a dual-layered hexagon. The boundary represents the collector venules (cv), and its electric resistance was set to 1 to 1/16 of the resisters arranged in the inner circuit representing the choriocapillaris. The voltage of the anode (*) was set at the center of the hexagon as 50 V. The cathode (dv) was arranged at the periphery of the hexagon representing the draining venules, and its voltage was set as 10 V. (**d**) Hexagons were arrayed as 5 × 5 to represent the choriocapillary bed. (**e**) Disconnecting an anode from one hexagon represents embolism of a single feeding arteriole. (**f**) Disconnecting anodes from a sector of hexagons represents embolism of a posterior ciliary artery.

For example (Fig. 6a), at point r, the sum of currents is zero. Because current (I) is equal to voltage (V)/resistance (R), the currents at node r can be written as equation (1).

1 $$({\rm{Vr}}-{\rm{Vp}})/{\rm{R}}1+({\rm{Vr}}-{\rm{Vq}})/{\rm{R}}2+({\rm{Vr}}-{\rm{Vs}})/{\rm{R}}3=0$$

In the same way, we can get more linear equations at any confluence points as many as unknown variables. These equations can be simplified for Vp = 0 and Vq = 10:

2 $$\begin{array}{l}{\rm{Node}}\,{\rm{r}};{\rm{Vr}}\times (\frac{1}{{\rm{R}}1}+\frac{1}{R2}+\frac{1}{R5})-\frac{Vs}{R5}=\frac{10}{R2}\\ {\rm{Node}}\,{\rm{s}};{\rm{Vs}}\times (\frac{1}{{\rm{R}}3}+\frac{1}{R4}+\frac{1}{R5})-\frac{Vr}{R5}=\frac{10}{R4}\end{array}$$

These linear equations make a matrix which composed of unknown voltage column and constants column:

3 $$\left[\begin{array}{cc}\left(\frac{1}{R1}+\frac{1}{R2}+\frac{1}{R5}\right) & -\frac{1}{R5}\\ -\frac{1}{R5} & \left(\frac{1}{R3}+\frac{1}{R4}+\frac{1}{R5}\right)\end{array}\right]\times \,[\begin{array}{c}Vr\\ Vs\end{array}]=\,[\begin{array}{c}\frac{10}{R2}\\ \frac{10}{R4}\end{array}]$$

Once after the equation matrix is completed, Gaussian elimination method can be used to get the inverse of the equation matrix. The product of the inverse matrix and constant column vector gives the solution vector of voltages.

The structures of the choriocapillaris bed were taken from corrosion cast study literature^7, 25^. A dual-layered hexagon with an internal grid composed of triangles was designed for the choriocapillaris lobule (Fig. 6). Each line of the grid has a resistance of 16 Ω, representing the capillary, and the boundary of the hexagon has a resistance of 1 to 16 Ω, representing the collector venule. The lumen of the vessels in the choriocapillaris plane measured 8–15 μm in the cats’ eyes^9^. The resistance was calculated based on the estimation that the diameter of the collector venule is between 1 × and 2 × that of the capillary; flow resistance is inversely proportional to the fourth power of the radius (r^4^) by Poiseuille’s equation. An anode representing the terminal arteriole was placed at the center of the hexagon, and cathodes representing the draining venules were connected at every other corner of the hexagon. The voltage was set as 51 V at the anode and 23 V at the cathodes to correspond with the intravascular pressure of the choroidal arteriole and venule in mmHg (equivalent to 69 and 31 cm H_2_O)^28^. A total 25 or 5 × 5 hexagons were arrayed to represent the choriocapillaris bed (Fig. 6c).

The flow of each vascular channel was calculated as an electric current, and the direction and amount were demonstrated by an arrow and its thickness, respectively. The average current in each hexagon was also calculated. Changes in electric currents were analyzed when changing the resistance of the hexagon boundary representing the collector venule. In this model, removing the anode connected to the center of one hexagon can simulate the occlusion of a single feeding arteriole (Fig. 6d), and multiple removals can simulate the occlusion of a PCA (Fig. 6e). Although the electric circuit model cannot calculate the actual flow in the choriocapillaris, the relative changes can be estimated compared with normal circulation.

## Electronic supplementary material

Supplement video
